# Reflections on Using the Eating Disorders Examination to Assess Eating Disorder Pathology in Queer Men

**DOI:** 10.1002/eat.24526

**Published:** 2025-08-13

**Authors:** Emma Austen, Jocelyn R. Clarke, Isabel Chua, Sarah Giles, Patrick Haylock, Imran M. Keshani, Po‐Han Kung, Elyse O’Loghlen, Scott Griffiths

**Affiliations:** ^1^ Melbourne School of Psychological Sciences University of Melbourne Melbourne Australia; ^2^ Faculty of Psychology, Counselling and Psychotherapy Cairnmillar Institute Hawthorn Australia; ^3^ School of Psychology Deakin University Burwood Australia; ^4^ Department of Psychological Sciences Swinburne University of Technology Hawthorn Australia

**Keywords:** eating disorder examination, eating disorders, men, qualitative, queer men

## Abstract

**Objective:**

Queer men face potent appearance‐related pressures that exacerbate their eating disorder risk. While the Eating Disorder Examination (EDE) is a widely used eating disorder assessment, queer men may experience unique motivations for disordered eating that may impact its administration in this population. To generate practical guidance for clinicians and researchers using the EDE, we qualitatively examined reflections from interviewers who administered the EDE to queer men.

**Method:**

Thirteen provisionally or generally registered psychologists administered the EDE to 179 queer men (*M*_age_ = 39.52, 84.36% with an eating disorder diagnosis, 87.15% identifying as gay) to assess their eligibility for a clinical trial of an eating disorder's intervention in Australia. Interviewers provided written reflections on their experience administering the EDE, which were analyzed with reflexive thematic analysis.

**Results:**

Interviewers noted that stringent norms around appearance, dieting, and exercise among queer men impaired men's insight into the harm caused by disordered behaviors, despite marked distress, and/or impairment. Usefully, interviewers noted the EDE helped men to gain insight into their disordered behaviors. Interviewers noticed that norms around dieting skewed what some participants perceived to be a “large” amount of food—this was useful context for interviewers to consider when distinguishing subjective from objective bulimic episodes.

**Discussion:**

Interviewers administering the EDE to queer men should be cognizant of subcultural appearance‐related pressures that may lead queer men to underreport impairment or to misjudge instances of their own overeating. Having this knowledge ahead of administering the EDE can equip interviewers to deliver this assessment accurately.

**Summary**

- We suspected that queer men experience unique motivations for, and presentations of, disordered eating that might impact the administration of the Eating Disorder Examination (EDE) in this population.
- To generate actionable recommendations for other researchers and clinicians using the EDE, we qualitatively examined interviewers' reflections on their experiences with administering the EDE to 179 queer (e.g., gay, bisexual) men as part of a clinical trial.
- Interviewers noted that the idealization of visibly muscular bodies within communities of queer men cultivates norms around adhering to strict diet and exercise regimes. These norms appeared to predispose some participants to engage in disordered eating behaviors and impaired their insight into the harm caused by these behaviors, despite marked distress and/or functional impairment. We highlight specific questions in the EDE that interviewers felt were useful for helping men to generate insight.
- Norms around dieting may have skewed what some participants perceived to be a “large” amount of food, which was useful context for interviewers to consider when distinguishing subjective from objective bulimic episodes. We highlight additional prompts that interviewers might use to guide their evaluation of objective bulimic episodes in this population and more broadly.

## 1 Introduction

The Eating Disorder Examination (EDE; Fairburn et al. 2014) is a semi‐structured interview historically referred to as a “gold standard” assessment for eating disorders (Rizvi et al. 2000; Weissman et al. 2016). The EDE produces frequency ratings for behavioral features of eating disorders, which can be used to generate DSM‐5 eating disorder diagnoses (American Psychiatric Association 2013). Beyond the EDE protocol paper (Fairburn et al. 2014), no literature has provided guidance on administering the EDE. To effectively deliver the EDE, interviewers should be trained in good interviewing technique (e.g., establishing rapport), and in concepts covered by the EDE (e.g., evaluating objective bulimic episodes; Fairburn et al. 2014). Understanding interviewers' experiences in administering the EDE can provide insight into the challenges that interviewers might encounter and generate practical guidance for those new to administering this protocol. Here, we qualitatively examine interviewers' reflections on administering the EDE to queer men (i.e., men with a sexual identity that is not exclusively heterosexual), who face potent appearance pressures (Bonell et al. 2023) that may engender unique considerations for the administration of the EDE.

### 1.1 Eating Disorders in Queer Men

Queer men's vulnerability to developing disordered eating warrants their prioritization in eating disorders research. The misconception that men rarely experience eating disorders has led to research in samples primarily comprised of women, and therefore eating disorder frameworks that mainly reflect women's experiences (Griffiths et al. 2013; Murray et al. 2017). Therefore, eating disorder assessments, including the EDE, may not adequately account for men's experiences (Darcy and Lin 2012). Compounding this, queer men are more likely to have a diagnosed eating disorder, to report restrictive eating, binge eating, and purging behaviors, relative to heterosexual men (Parker and Harriger 2020). This vulnerability has been attributed to the minority stressors that queer men face (Feldman and Meyer 2007). For example, societal stereotypes that frame queer men as “effeminate” may motivate men to engage in disordered eating and exercise behaviors to attain a “masculine” (i.e., lean, muscular) physique (Alexander et al. 2024). These pressures are reinforced within communities of queer men: relative to heterosexual men, queer men face greater pressure to be visibly muscular from the media, partners, and other queer men (Jankowski et al. 2014).

Variations in appearance ideals across subcultures of queer men may engender unique presentations of, and motivations for, disordered eating. In Fogarty and Walker's (2022) study of eating pathology among 241 gay men in the US, 44% of the sample reported identifying as a member of a subculture with its own appearance ideals. Examples of such *subcultural appearance identities* include *twinks*, who tend to be younger and thinner, *bears*, who tend to be larger‐bodied with body hair, and *jocks*, who have an “athletic” build (Fogarty and Walker 2022). Men who identified as twinks, jocks, and bears reported greater muscularity‐oriented eating pathology relative to those without a subcultural appearance identity, suggesting that having a subcultural appearance identity may elevate queer men's risk for muscularity‐oriented eating pathology. Thus, motivations for disordered eating may vary among queer men, both because queer men (on average) report greater investment in appearance relative to heterosexual men, and because the nature of appearance ideals varies *within* subcultures of queer men.

### 1.2 The Current Study: The Value of Interviewer Perspectives on Administering the EDE to Queer Men

We suspected that the unique ways eating disorders can present among queer men, and the unique sociocultural factors that contribute to their development, might require interviewers to modify their administration of the EDE. For example, interviewers might need to tailor their approach to prepare for the fact that men are less likely to recognize their behavior as disordered (Murray et al. 2017). Further, interviewers might need to know that queer men can report motivations for behaviors that are tied to appearance ideals perpetuated within specific subcultures (Fogarty and Walker 2022). In this study, we qualitatively examine interviewers' reflections on administering the EDE to 179 queer men. In summarizing these reflections, we generate practical insights to guide researchers and clinicians using the EDE in this population, and in the broader eating disorder population.

## 2 Method

### 2.1 Clinical Trial Information and Procedure

The reflections in this paper come from interviewers who worked on a randomized controlled trial (RCT) of an eHealth intervention targeting eating disorder symptoms (trial registration ID: ACTRN12621001244897). The RCT comprises two sub‐studies, which were conducted independently. The reflections in this paper come from the sub‐study that investigated the intervention's efficacy among cisgender queer men. The University of Melbourne Human Research Ethics Committee (IDs: 30177, 13,046) approved this study's protocol.

We recruited participants primarily via advertisements on smartphone dating/hook‐up applications targeted at queer men (e.g., Grindr) and social media (e.g., Instagram). Advertisements included a link to a Qualtrics survey that screened for participant eligibility. Participants who self‐reported a non‐heterosexual sexual identity, were over 18 years old, reported a history of dieting or body image concerns, and did not self‐report a current diagnosis of Avoidant/Restrictive Food Intake Disorder (ARFID), anorexia nervosa, or atypical anorexia nervosa were eligible to proceed to the EDE, which was the final stage in eligibility screening.

#### 2.1.1 EDE Interviews

EDE interviews were conducted remotely via phone or Zoom. Interviewers used the 17th edition of the EDE (Fairburn et al. 2014). Interviews were not recorded. Our interviewing team comprised 13 provisional or registered psychologists who had completed or were currently undertaking an accredited clinical psychology program (e.g., Masters or Doctorate) at an Australian university and had at least 6 months of placement experience. The first and senior authors recruited interviewers via their own professional connections and email callouts to convenors of accredited Australian clinical psychology programs. Table 1 presents descriptive information about interviewers.

**TABLE 1:** Interviewer Characteristics (*N* = 13).

Characteristic	*n* (%)
Psychologist registration^a^	
Provisional registration	8 (61.54)
General registration	5 (38.46)
Prior experience administering EDE before this project^a^	
Yes	5 (38.46)
No	8 (61.54)
Cumulative experience working in clinical settings (e.g., placement as part of degree, or practice as registered psychologist)^a^	
6–11 months	6 (46.15)
1–2 years	3 (23.08)
> 2 years	4 (30.77)

^a^ Status of registration and level of experience as of March 2024 (start of recruitment for RCT).

Our interviewers were already trained to administer the EDE or underwent training as part of their involvement in the trial. EDE training involved: (i) reading the EDE protocol paper (Fairburn et al. 2014) and literature on eating disorders in men (e.g., Strother et al. 2012), (ii) attending a training session led a clinical psychologist with substantial expertise in administering the EDE and training other psychologists to administer the EDE, and (iii) attending team meetings. Team meetings involved discussing experiences with the EDE and coming to consensus around scoring decisions. The senior author, who has substantial experience in the diagnosis and phenomenology of eating disorders in men, briefed interviewers on manifestations of eating disorders in men, with queer men as a central focus. Interviewers received training in cultural competency as part of their accredited Masters or Doctoral clinical psychology programs.

Interviewers reflected on their experiences with the specific goal of informing an article that would be useful for others administering the EDE. Interviewers were provided prompts to loosely guide their reflections (see Supporting Information) but were encouraged to reflect outside of these prompts. Interviewers wrote their reflections in a shared document immediately after interviews and in cases where interviewees did not attend their appointment.

#### 2.1.2 Interviewees

In total, 179 men completed an EDE interview. Tables 2 and 3 summarize interviewee characteristics.

**TABLE 2:** Interviewee demographic characteristics (*N* = 179).

Characteristic	Category	*n* (%)
Sexual identity	Gay	156 (87.15)
	Bisexual	16 (8.94)
	Pansexual	3 (1.68)
	Another sexual identity	2 (1.12)
	Prefer not to say	2 (1.12)
Gender identity	Cis‐gender man	179 (100.00)
Eating disorder diagnosis^a^	Other specified eating or eating disorder	64 (35.75)
	Sub‐threshold bulimia nervosa	30 (16.76)
	Sub‐threshold binge eating disorder	22 (12.29)
	No specifier provided	12 (6.70)
	Binge eating disorder	38 (21.23)
	Bulimia nervosa	19 (10.61)
	Atypical anorexia nervosa	15 (8.38)
	Unspecified feeding or eating disorder	14 (7.82)
	Diagnosis uncategorised due to server‐side error in data collection	1 (0.56)
	No eating disorder diagnosis	28 (15.64)
Race	White	84 (46.93)
	Asian	11 (6.15)
	Hispanic, Latino, or Spanish	5 (2.79)
	South Asian	4 (2.23)
	Middle Eastern or North African	5 (2.79)
	Multiple racial identities	6 (3.35)
	Aboriginal or Torres Strait Islander	2 (1.12)
	Pacific Islander	1 (0.56)
	Prefer not to say	1 (0.56)
	Missing	60 (33.52)

	M (SD)	Possible range	Observed range
Age	39.52 (12.30)		18–70
Socioeconomic status^b^	7.15 (1.67)	1–10	1–10

^a^ Diagnoses were generated in line with the criteria stipulated in Fairburn et al. (2014), 4–7. OSFED specifiers were assigned where: (1) for atypical anorexia nervosa, all DSM‐5 criteria for anorexia nervosa were met, except the participant's BMI was ≥ 18.5 kg/m^2^, (2) for sub‐threshold binge eating disorder, objective binge episodes were present but fewer than 12 times in the preceding 3 months, and (2) for sub‐threshold bulimia nervosa, objective binge eating and compensatory behaviors were present but less frequently than specified in the DSM‐5 criteria for bulimia nervosa (i.e., less than 12 objective binge episodes in the preceding 3 months, and/or engaging in compensatory behaviors less than once per week and for less than 3 months).

^b^ *n* = 121. Participants self‐reported their perceived socioeconomic status on a hypothetical ladder (1 = lowest social status, 10 = highest social status; Adler et al. 2000).

**TABLE 3:** EDE global and subscale scores in participants with and without an eating disorder diagnosis.

EDE subscale	With diagnosis (*n* = 151 [84.36%])		Without diagnosis (*n* = 28 [15.64%])	
	M (SD)	Observed range	M (SD)	Observed range
Global	3.05 (0.90)	0.96–5.51	1.88 (0.79)	0.54–3.71
Weight concern	3.68 (1.21)	0.00–6.00	2.49 (1.10)	0.00–4.75
Shape concern	3.91 (1.09)	1.25–6.00	2.73 (1.27)	0.62–5.88
Eating concern	1.86 (1.29)	0.00–5.75	0.66 (0.59)	0.00–2.00
Restraint	2.75 (1.22)	0.00–5.40	1.64 (1.23)	0.00–4.40

*Note*: The “with diagnosis” group includes participants with a sub‐threshold diagnosis and those who met the full criteria for an eating disorder diagnosis.

### 2.2 Data Analysis

We used reflexive thematic analysis to generate themes from interviewer reflections. Our analysis was informed by pragmatism, a research paradigm that aims to generate knowledge with practical applicability (Kaushik and Walsh 2019). The first author led the data analysis. EA followed Braun and Clarke's (2022) recommended steps to reflexive thematic analysis, where patterns in data are grouped into “codes,” and then into broader themes that communicate the shared sentiment of these groups of codes. She generated the themes inductively (i.e., themes were driven by the data, not theory) and semantically (i.e., themes captured the explicit, not implicit, meaning of the data). She used NVivo 14 to code the data.

EA was a project manager for the trial and did not administer the EDE interviews. She consulted co‐authors who were part of the interviewing team (J.R.C., I.C., S.G. [second author], P.H., I.M.K., P.K., E.O.) throughout the analysis to confirm that themes were representative of interviewer experiences and maximize their practical applicability. After generating the first round of themes, EA sought feedback from these co‐authors and refined themes where required. The structure of the themes was altered once more in response to peer review feedback; these changes improved the clarity of the analysis while retaining the key information communicated throughout. All authors approved the final themes presented in this paper.

## 3 Results

Here, we present our themes with an integrated interpretation of these themes in the context of existing literature. Consistent with existing literature (e.g., Bonell et al. 2023), interviewees frequently referenced subcultural pressures around appearance, diet, and exercise that can be perpetuated among queer men:

Many participants reported … strict calorie tracking, intermittent fasting, and other diets … [they] were driven by the desire to achieve an idealized shape [lean, muscular] prevalent within certain LGBTQIA+ subcultures and perceived these behaviors as “healthy” and the “right thing to do.” …

We generated three themes that encompass interviewers' reflections on the impact of these subcultural norms around appearance, diet, and exercise on the EDE's administration. Interviewers noted that these norms may: (1) shape men's motivations for eating and exercise behaviors; (2) impact men's insight into the presence and impact of disordered behaviors; and (3) complicate assessment of objective bulimic episodes. Table 4 summarizes our themes alongside recommendations for interviewers using the EDE.

**TABLE 4:** Key findings from our study with corresponding recommendations for clinicians and researchers using the EDE in their work.

Theme/subtheme	Recommendation for EDE interviewers
Sub‐cultural norms around appearance, diet and exercise among queer men may shape men's motivations for engaging in disordered eating and exercise behaviors	Be aware of unique considerations for eating disorders in men ahead of commencing interviews—e.g., motivations for behaviors and potential for reduced insight.
Sub‐cultural norms around appearance, diet and exercise among queer men may impact men's insight into the presence and impact of disordered behaviors	Reflect on norms around appearance, diet, and exercise among queer men; how these may differ from the interviewers' own perceptions; and how such difference may impact interviewers' judgments on what behaviors are considered disordered.
Specific questions in the EDE can aid insight generation	Men may require extra prompting throughout the interview to help generate insight. Questions around importance of weight and shape (Fairburn et al. 2014, 28–29) and dietary rules (Fairburn et al. 2014, 9) can aid insight generation in cases where men cannot articulate the presence or extent of impairment caused by behaviors.
Prescription of weight loss regimes to participants by their healthcare providers might reflect the underrecognition of disordered eating in queer men, and complicate EDE assessment	Ask whether participants have been prescribed weight loss medications (e.g., semaglutide) or diets by healthcare providers. Enquire as to whether participants are taking medications such as semaglutide as prescribed, or may be misusing them (e.g., query around unusually rapid or excessive weight loss. See: National Eating Disorder Collaboration 2025)
Sub‐cultural norms around appearance, diet and exercise among queer men complicated interviewers' assessment of objective versus subjective bulimic episodes.	Be aware that sub‐cultural norms around dieting and weight loss may inform participants' assessment of what other people might consider having overeaten (e.g., “Have there been any times when you have felt that you have eaten an ordinary amount of food but others might have regarded you as having overeaten?”; Fairburn et al. 2014, 13). Suggestions for aiding evaluation of episodes of overeating: ○ Ask participants who they are thinking of when comparing their experiences of overeating to how they may be perceived by others. ○ Ask participants to think of an “average” person rather than their immediate social circle, in case subcultural norms around dieting and exercise are skewing perceptions about what might be a “typical” amount of food. ○ Ask participants to describe their instances of “overeating” in a standard “unit”—for example, number of plates, or ask to describe what the plate of food looked like Questions on overeating can be difficult for both interviewers and interviewees to navigate. Practice these questions ahead of time, and/or preface the scope of these questions to participants so that they are easier to navigate. For example: *“Some of these questions might sound repetitive …”*

### 3.1 Theme 1: Subcultural Norms Around Appearance, Diet, and Exercise May Shape Queer Men's Motivations for Disordered Eating and Exercise Behaviors

Under this theme, interviewers reflected on how norms around dieting and exercise among queer men may shape men's motivations to engage in disordered behaviors. For example, one interviewer noted that interviewees often reported these norms as a motivation for restrictive eating:

[for] questions surrounding “consciously restricting food for another reason such as to give them a sense of control or feeling in control in general” … [participants] were almost exclusively motivate[d] by … sub‐cultural norms around dieting/weight. This … differs from my experiences interviewing women and neurodivergent individuals who regularly endorse that a motivation for restricting food is that it gives them a sense of control which ameliorates anxiety which [then] reinforces ED behaviors.

Contrastingly, another interviewer flagged that some of their participants *did* cite a sense of control as a motivation for their behaviors that was linked to a desire to adhere to masculine norms:

… a couple of participants explicitly link[ed] this sense of control to societal expectations of masculinity (e.g., men should have a strong will and stay in control of their actions). While their restriction/strict exercise regimen[s] are motivated by appearance‐centric reasons, their experienced distress … [was] exacerbated by the perception of failing at those masculine expectations.

These reflections illustrate how masculine norms shape the presentation of eating disorders in queer men. The societal privileging of hegemonically masculine (i.e., White, cis‐gender, heterosexual) identities gives rise to pejorative stereotypes that queer men are “effeminate”/unmasculine (Connell and Messerschmidt 2005). Thus, visibly masculine (i.e., muscular) bodies may be idealized within communities of queer men because they contradict these stereotypes (Alexander et al. 2024). These pressures cultivate an environment where queer men are motivated to engage in disordered eating and exercise to align themselves with masculine ideals (Bonell et al. 2023). Our interviewer reflections suggest that masculine norms may facilitate disordered eating among queer men by: (i) shaping esthetic goals and the eating and exercise behaviors employed in pursuit of them, and (ii) driving men's desire to adhere to masculine norms like self‐control by following rigid ways of dieting and exercising.

Adequate content knowledge, including of the potential motivations underlying disordered behaviors, is central to effectively administering the EDE (Fairburn et al. 2014). Those who are new to administering the EDE among queer men should understand that men's engagement in disordered behaviors may be motivated by norms around dieting and exercise within communities of queer men, along with a desire to adhere to masculine norms by exhibiting self‐control around diet and exercise.

### 3.2 Theme 2: Norms Around Appearance, Diet and Exercise Among Queer Men May Impact Men's Insight Into the Presence and Impact of Disordered Behaviors

This theme encompasses interviewers' reflections on men's insight into the presence and impact of their disordered eating behaviors. One interviewer considered that the “normalcy” of restrictive dieting or exercise within communities of queer men might impair some men's insight into the harm of these behaviors.

[Among queer men] … there is a strong emphasis on weight loss and fitness that can overshadow conversations about disordered eating. This rhetoric often blinds individuals to the potential harm of their behaviors.

This observation aligns with evidence that men are less likely to recognize their behaviors as eating disorder symptoms because of stereotypes that frame eating disorders as something only women experience (Räisänen and Hunt 2014). The intra‐minority stressors experienced by queer men, including cultural pressures to be muscular via strict diet and exercise (Pachankis et al. 2020), may contribute to queer men overlooking the distress and functional impairment resulting from these behaviors and impair their recognition of these behaviors as disordered. We generated two subthemes that illustrate ways to account for this reduced insight via the EDE and one potential consequence of this reduced insight, respectively: (i) Specific questions in the EDE can aid insight generation, and (ii) Prescription of weight loss regimes to participants by their healthcare providers might reflect the underrecognition of disordered eating in men and complicate EDE assessment.

#### 3.2.1 Specific Questions in the EDE Can Aid Insight Generation

In their qualitative investigation of the phenomenology of muscle dysmorphia, Martenstyn et al. (2022) note follow‐up questions might be required in clinical interviews with men to gain a full understanding of their experiences. Similarly, some interviewers in our study noted that participants *could* identify the emotional impact of behaviors when prompted by specific questions in the EDE:

… participants I interviewed [could] very clearly describe the emotional impact of not following their ideal regimen … the questions around perceived sense of wrong‐doing, or distress around binging/eating were helpful to evaluate the severity of disordered eating patterns.

Thus, norms around diet and exercise may impact *some* men's ability to recognize behaviors as disordered. In these circumstances, interviewers might find specific questions in the EDE (e.g., perceived guilt about eating; Fairburn et al. 2014, 18) helpful for aiding participants to recognize the presence or extent of impairment.

Relatedly, the most common sentiment across interviewer reflections was that the EDE helped men to develop insight: “*… the EDE seemed to provide the opportunity for … men to develop insight, acknowledge their problems and begin the process of help seeking.”* Thus, the EDE enabled men to reflect on the potential harm of their behaviors, which is otherwise difficult in a cultural context where disordered eating among men is underrecognized (Bonell et al. 2023). Two interviewers identified questions that were helpful for insight generation:

… questions gauging the importance of weight vs shape/figure in self‐evaluation [Fairburn et al. 2014, 28–29] … highlight[ed] the extent of [interviewees'] struggles with eating and self‐perception.

… the dietary rules question: “Have they been definite rules or general guidelines?” [Fairburn et al. 2014, 9] … seemed to be really helpful for insight building …

These reflections highlight that some queer men experiencing disordered eating may not have insight into the impairment caused by disordered behaviors, and that the EDE can be helpful for facilitating insight in these cases. Existing studies with samples of queer men predominantly administer self‐report measures like the EDE questionnaire (EDE‐Q; Nagata et al. 2024), which, although less time‐intensive to administer, may not offer the same level of insight generation as a semi‐structured interview, where open‐ended questions enable elaboration.

#### 3.2.2 Prescription of Weight Loss Regimes to Participants by Their Healthcare Providers Might Reflect the Underrecognition of Disordered Eating in Men, and Complicate EDE Assessment

Several interviewees of higher weights had been prescribed weight loss diets or drugs, including glucagon‐like peptide‐1 receptor agonists (GLP‐1 RAs) such as semaglutide/Ozempic, by their healthcare providers. We argue that this reflects a lack of recognition from both participants *and* their healthcare providers of the presence and impact of disordered eating among queer men, particularly those with higher body weights. Eating disorders in people of higher weights are underdiagnosed and undertreated, despite comprising over half of Australians with an eating disorder (da Luz et al. 2017). This is a product of weight stigma: people of higher weights are frequently prescribed weight loss regimes for their ailments without first being screened for disordered eating (Puhl and Suh 2015; Tomiyama et al. 2018). Compounding this further, queer men of higher body weights simultaneously grapple with broader societal weight stigma *and* potent appearance norms perpetuated within communities of queer men. These pressures exacerbate their vulnerability to engaging with disordered eating and to seek weight loss treatments (Austen et al. 2020; Levinson et al. 2024). Together, the lack of recognition and treatment seeking for disordered eating among men (Murray et al. 2017), coupled with weight stigma, may facilitate the prescription of weight loss medications to queer men of higher weights without prior eating disorders screening.

Notably, several interviewers felt that the prescription of weight loss drugs or diets to men in the current sample complicated their assessment of preoccupation or restriction in the EDE:

… a participant [who was using Ozempic] reported reduced symptoms, complicating the evaluation of behaviors like restriction or preoccupation with food that were present before starting the medication.

… Some individuals report[ed] that particular diets (e.g., intermittent fasting) have been recommended by healthcare providers, [it is] therefore difficult to assess whether these are relevant to [questions asking about regularity of eating meals and snacks in the past four weeks] as “disordered”.

Thus, EDE interviewers should be aware that people of higher weights may be prescribed weight loss regimes, including medications and/or diets, by their health practitioners; and that this can complicate the evaluation of the presence of restriction or frequency of preoccupation with weight and shape in the EDE.

### 3.3 Theme 3: Subcultural Norms Around Diet and Exercise Can Complicate Assessment of Objective Versus Subjective Bulimic Episodes

This theme encompasses interviewers' reflections on the challenges associated with evaluating objective bulimic episodes using the EDE. The EDE protocol specifies two defining characteristics of objective bulimic episodes: (i) a “loss of control”, and (ii) “the consumption of what would generally be regarded as a ‘large’ amount of food” (Fairburn et al. 2014, 11). To assess this, respondents are asked to use social context to classify instances of overeating (e.g., “Have there been any times when you have felt that you have eaten an ordinary amount of food but others might have regarded you as having overeaten?”; Fairburn et al. 2014, 13).

Interviewers noted that sub‐cultural norms around diet and exercise among queer men complicated their assessment of what is generally regarded as a “large amount of food”. One interviewer noted:

… using the perspectives of what [an interviewee's] peers consider as “normal” to judge whether what [they've] eaten is abnormal might distort [an interviewer's] ability to reach some sort of “objective” assessment of whether the person binge eats or not …

Speaking to how interviewers might adjust their approach to the EDE to account for this potential subjectivity, this interviewer added:

It might be helpful to enquire who the participant is thinking about when they are comparing their episodes of overeating to how these might be viewed by others …[or] to use language like what an “average person” would think rather than relying on the person's immediate social circle.

Speaking to broader concerns with the administration of the EDE, another interviewer noted that they found it challenging to frame questions on bulimic episodes in a way that made it clear what information participants should be providing:

The trickiest part of the EDE is the section on bulimic episodes and other episodes of overeating. Participants often get confused about what they're supposed to report (e.g., including subjective episodes in their responses to questions on objective episodes) …

Providing insight into how they navigated these challenges, they added: “I've needed to be extra clear in highlighting the parameters of questions in this section to make sure that participants are making correct reports.”

Together, the reflections in this theme corroborate existing critiques of the complexity of evaluating bulimic episodes in eating disorders' assessment (Latner and Clyne 2008; Mond et al. 2010) and highlight that these questions are difficult to navigate for both interviewers *and* respondents. These difficulties may be compounded in the administration of the EDE to queer men experiencing disordered eating, specifically, who may require extra prompting from interviewers to determine whether self‐reported episodes of overeating reflect an objectively large amount of food. Our findings suggest there is scope for further prompts in the EDE to help interviewers in distinguishing subjective from objective bulimic episodes (Table 4 provides suggestions). Potential options offered by our interviewers' reflections are to inquire about who the interviewee is thinking about when answering questions about episodes of overeating, or to provide them a reference point (e.g., an “average person”). It is important to evaluate whether a concurrent loss of control is present in this context, as men are more likely to report deliberately consuming an objectively large amount of food to enhance their muscularity (i.e., “bulking”; Martenstyn et al. 2022). Thus, eating an objectively large amount of food when aiming to gain muscle may not constitute a bulimic episode unless a clear loss of control is present. Interviewers might practice their delivery of these questions ahead of time; clear and effective delivery of the EDE ensures that participants understand what they are being asked, and therefore that the data collected is as reliable as possible (Fairburn et al. 2014).

## 4 Discussion

We qualitatively examined interviewer's reflections on administering the EDE among queer men to generate practical guidance for clinicians and researchers using this assessment in their own work. Our interviewer reflections indicate that there are unique considerations for administering the EDE to queer men. Potent norms around appearance, diet, and exercise among queer men might cultivate an environment where engaging in disordered eating and exercise to attain a muscular physique is normalized. As a result, queer men may have reduced insight into the presence and/or negative impacts of these disordered behaviors. These norms may also shift the reference points men experiencing disordered eating use in describing instances of “overeating” and impact interviewers' assessment of objective bulimic episodes.

### 4.1 Implications and Future Directions

Our recommendations (Table 4) can support those new to administering the EDE, particularly among queer men. For example, people who train new EDE interviewers might provide guidance around how to navigate evaluating bulimic episodes and/or flag questions that are useful for generating insight in queer men. This paper may be recommended reading for new interviewers to consult as part of their training. Our findings might also inform future adaptations of the EDE—for example, through the inclusion of additional prompts to help interviewers evaluate bulimic episodes. Adaptations could provide explicit guidance around evaluating bulimic episodes among men, who are more likely to report deliberately consuming an objectively large amount of food to enhance their muscularity (i.e., “bulking”; Martenstyn et al. 2022). Thus, adaptations might emphasize that such instances may not constitute a bulimic episode unless a clear loss of control is present. Usefully, there is an existing adaptation of the EDE for generating ARFID diagnoses (Schmidt et al. 2019). We hope that our recommendations can similarly guide future adaptations of the EDE and aid researchers and clinicians to deliver this tool confidently and accurately.

More broadly, the presence of participants who were prescribed weight loss regimes, including GLP‐1 RA medications, in our sample reiterates a need for health professionals to receive adequate training around eating disorders, and to screen for eating disorders prior to recommending weight management. This is particularly prudent among groups who are vulnerable to experiencing disordered eating but among whom these behaviors are underrecognized, including queer men. The National Eating Disorders Collaboration has two useful resources for this purpose: (i) guidelines on the management of eating disorders for people with higher weights (Ralph et al. 2022); and (ii) a factsheet on potential risks associated with using GLP‐1 RAs in the context of eating disorders (National Eating Disorder Collaboration 2025).

We acknowledge key aspects of the research team's positionalities that may have shaped this research. The first author, who led the data analysis, did not conduct the EDE interviews, which might have created “blind spots” in her understanding of what reflections were especially useful to highlight for clinicians administering the EDE. However, she consistently communicated with the interviewing team to ensure that the themes generated were representative of their reflections and maximally practical. Second, the interviewing team varied in their sexual identities and prior experience using the EDE. Some team members identified as queer men—these interviewers acknowledge that their shared identity with interviewees may have (1) facilitated greater rapport with interviewees and/or (2) led these interviewers to perceive some eating or exercise patterns as “normal”, potentially resulting in oversight of milder disordered eating patterns. To account for this, these interviewers concentrated on participants' subjective experience of dysfunction and distress when evaluating symptom severity. Similarly, some interviewers reflected on how their ethnicity and/or cultural background—sometimes inferred by cues like their skin color or name—might have shaped rapport developed with participants; for example, assumed shared cultural experiences might have aided rapport. Lastly, interviewers underwent comprehensive training to ensure consistency across interviewers. However, 62% of interviewers had not used the EDE prior to this study, which may have produced some inconsistency in EDE ratings. This variation in experience is advantageous for generating practical insights for other researchers and clinicians with similarly varied experience. Nonetheless, future studies could assess inter‐rater reliability to inform stronger inferences around the generalizability of findings.

Future research can expand on the scope of our findings by employing similar methods in diverse samples. For example, interviewees in this study were cis‐gender men who predominantly identified as gay and White. Further, our study did not include men with ARFID, atypical anorexia nervosa, or anorexia nervosa. Motivations for, and presentations of, eating disorders vary across demographic groups—for example, people with intersecting marginal identities (e.g., across ethnicity, gender, and sexual identity) face unique, intersecting cultural pressures that compound their risk of developing disordered eating (Burke et al. 2020). As highlighted in our study, such cultural considerations can be relevant for interviewers to consider when administering the EDE. Future studies might explore the logistics of administering the EDE across a range of groups (e.g., gender and/or sexual identities, eating disorder diagnoses, and intersectional identities) to determine what factors or challenges interviewers might need to account for when administering the EDE in different contexts.

## 5 Conclusion

Our findings underscore the importance of exploring clinician and researcher experiences with administering eating disorder assessments in groups like queer men, among whom these assessments were not originally developed and validated (Darcy and Lin 2012). Future research can continue to identify the practical challenges of administering the EDE to prepare researchers and clinicians to deliver this tool accurately and strengthen its reliability.

## Author Contributions

**Emma Austen:** conceptualization, investigation, methodology, formal analysis, project administration, supervision, writing – original draft, writing – review and editing, visualization, data curation. **Jocelyn R. Clarke:** investigation, writing – review and editing. **Isabel Chua:** investigation, writing – review and editing. **Sarah Giles:** investigation, writing – review and editing. **Patrick Haylock:** investigation, writing – review and editing. **Imran M. Keshani:** investigation, writing – review and editing. **Po‐Han Kung:** investigation, writing – review and editing. **Elyse O’Loghlen:** investigation, writing – review and editing. **Scott Griffiths:** conceptualization, methodology, supervision, project administration, resources, writing – review and editing, funding acquisition.

## Conflicts of Interest

The authors declare no conflicts of interest.

## Supporting information


**Data S1:** Supporting Information.

## Data Availability

The data that support the findings of this study are available on request from the corresponding author. The data are not publicly available due to privacy or ethical restrictions.

## References

[eat24526-bib-0001] Adler, N. E. , E. S. Epel , G. Castellazzo , and J. R. Ickovics . 2000. “Relationship of Subjective and Objective Social Status With Psychological and Physiological Functioning: Preliminary Data in Healthy, White Women.” Health Psychology 19, no. 6: 586–952. 10.1037/0278-6133.19.6.586. 11129362

[eat24526-bib-0002] Alexander, T. , C. B. Burnette , H. Cory , S. McHale , and M. Simone . 2024. “The Need for More Inclusive Measurement to Advance Equity in Eating Disorders Prevention.” Eating Disorders 32, no. 6: 798–816. 10.1080/10640266.2024.2328460. 38488765 PMC11401964

[eat24526-bib-0003] American Psychiatric Association . 2013. Diagnostic and Statistical Manual of Mental Disorders. 5th ed. American Psychiatric Association. 10.1176/appi.books.9780890425596.

[eat24526-bib-0004] Austen, E. , K. H. Greenaway , and S. Griffiths . 2020. “Differences in Weight Stigma Between Gay, Bisexual, and Heterosexual Men.” Body Image 35: 30–40. 10.1016/j.bodyim.2020.08.002. 32829093

[eat24526-bib-0005] Bonell, S. , M. J. Wilson , S. Griffiths , S. M. Rice , and Z. E. Seidler . 2023. “Why Do Queer Men Experience Negative Body Image? A Narrative Review and Testable Stigma Model.” Body Image 45: 94–104. 10.1016/j.bodyim.2023.02.005. 36867966

[eat24526-bib-0006] Braun, V. , and V. Clarke . 2022. “Conceptual and Design Thinking for Thematic Analysis.” Qualitative Psychology 9, no. 1: 3–26. 10.1037/qup0000196.

[eat24526-bib-0007] Burke, N. L. , L. M. Schaefer , V. M. Hazzard , and R. F. Rodgers . 2020. “Where Identities Converge: The Importance of Intersectionality in Eating Disorders Research.” International Journal of Eating Disorders 53, no. 10: 1605–1609. 10.1002/eat.23371. 32856342 PMC7722117

[eat24526-bib-0008] Connell, R. W. , and J. W. Messerschmidt . 2005. “Hegemonic Masculinity: Rethinking the Concept.” Gender & Society 19, no. 6: 829–859. 10.1177/0891243205278639.

[eat24526-bib-0009] da Luz, F. Q. , A. Sainsbury , H. Mannan , S. Touyz , D. Mitchison , and P. Hay . 2017. “Prevalence of Obesity and Comorbid Eating Disorder Behaviors in South Australia From 1995 to 2015.” International Journal of Obesity 41, no. 7: 1148–1153. 10.1038/ijo.2017.79. 28337025

[eat24526-bib-0010] Darcy, A. M. , and I. H.‐J. Lin . 2012. “Are we Asking the Right Questions? A Review of Assessment of Males With Eating Disorders.” Eating Disorders 20, no. 5: 416–426. 10.1080/10640266.2012.715521. 22985238

[eat24526-bib-0011] Fairburn, C. G. , Z. Cooper , and M. O'Connor . 2014. “Eating Disorder Examination (17.0D).” In Cognitive Behavior Therapy and Eating Disorders, edited by C. G. Fairburn , 265–308. Guilford Press. https://www.cbte.co/download/ede‐17‐0d/.

[eat24526-bib-0012] Feldman, M. B. , and I. H. Meyer . 2007. “Eating Disorders in Diverse Lesbian, Gay, and Bisexual Populations.” International Journal of Eating Disorders 40, no. 3: 218–226. 10.1002/eat.20360. 17262818 PMC2080655

[eat24526-bib-0013] Fogarty, S. M. , and D. C. Walker . 2022. “Twinks, Jocks, and Bears, Oh My! Differing Subcultural Appearance Identifications Among Gay Men and Their Associated Eating Disorder Psychopathology.” Body Image 42: 126–135. 10.1016/j.bodyim.2022.05.010. 35700650

[eat24526-bib-0014] Griffiths, S. , S. B. Murray , and S. Touyz . 2013. “Disordered Eating and the Muscular Ideal.” Journal of Eating Disorders 1: 15. 10.1186/2050-2974-1-15. 24999396 PMC4081800

[eat24526-bib-0015] Jankowski, G. S. , H. Fawkner , A. Slater , and M. Tiggemann . 2014. ““Appearance Potent”? A Content Analysis of UK Gay and Straight Men's Magazines.” Body Image 11, no. 4: 474–481. 10.1016/j.bodyim.2014.07.010. 25129685

[eat24526-bib-0016] Kaushik, V. , and C. A. Walsh . 2019. “Pragmatism as a Research Paradigm and Its Implications for Social Work Research.” Social Sciences 8, no. 9: 255. 10.3390/socsci8090255.

[eat24526-bib-0017] Latner, J. D. , and C. Clyne . 2008. “The Diagnostic Validity of the Criteria for Binge Eating Disorder.” International Journal of Eating Disorders 41, no. 1: 1–14. 10.1002/eat.20465. 17922537

[eat24526-bib-0018] Levinson, J. A. , S. Kinkel‐Ram , B. Myers , and J. M. Hunger . 2024. “A Systematic Review of Weight Stigma and Disordered Eating Cognitions and Behaviors.” Body Image 48: 101678. 10.1016/j.bodyim.2023.101678. 38278088 PMC11180546

[eat24526-bib-0019] Martenstyn, J. A. , S. Maguire , and S. Griffiths . 2022. “A Qualitative Investigation of the Phenomenology of Muscle Dysmorphia: Part 1.” Body Image 43: 486–503. 10.1016/j.bodyim.2022.10.009. 36356368

[eat24526-bib-0020] Mond, J. M. , J. D. Latner , P. H. Hay , C. Owen , and B. Rodgers . 2010. “Objective and Subjective Bulimic Episodes in the Classification of Bulimic‐Type Eating Disorders: Another Nail in the Coffin of a Problematic Distinction.” Behaviour Research and Therapy 48, no. 7: 661–669. 10.1016/j.brat.2010.03.020. 20434132

[eat24526-bib-0032] Murray, S. B. , J. M. Nagata , S. Griffiths , et al. 2017. “The Enigma of Male Eating Disorders: A Critical Review and Synthesis.” Clinical Psychology Review 57: 1–11. 10.1016/j.cpr.2017.08.001. 28800416

[eat24526-bib-0021] Nagata, J. M. , E. Stuart , J. O. Hur , et al. 2024. “Eating Disorders in Sexual and Gender Minority Adolescents.” Current Psychiatry Reports 26, no. 7: 340–350. 10.1007/s11920-024-01508-1. 38829456 PMC11211184

[eat24526-bib-0022] National Eating Disorder Collaboration . 2025. Eating Disorders and Glucagon‐Like Peptide‐1 Receptor Agonists (GLP‐1 RAs). https://nedc.com.au/assets/Fact‐Sheets/NEDC‐Eating‐Disorders‐GLP‐1‐RAs‐Factsheet.pdf.

[eat24526-bib-0023] Pachankis, J. E. , K. A. Clark , C. L. Burton , J. M. W. Hughto , R. Bränström , and D. E. Keene . 2020. “Sex, Status, Competition, and Exclusion: Intraminority Stress From Within the Gay Community and Gay and Bisexual Men's Mental Health.” Journal of Personality and Social Psychology 119, no. 3: 713–740. 10.1037/pspp0000282. 31928026 PMC7354883

[eat24526-bib-0024] Parker, L. L. , and J. A. Harriger . 2020. “Eating Disorders and Disordered Eating Behaviors in the LGBT Population: A Review of the Literature.” Journal of Eating Disorders 8, no. 1: 51. 10.1186/s40337-020-00327-y. 33088566 PMC7566158

[eat24526-bib-0025] Puhl, R. , and Y. Suh . 2015. “Stigma and Eating and Weight Disorders.” Current Psychiatry Reports 17, no. 3: 552. 10.1007/s11920-015-0552-6. 25652251

[eat24526-bib-0026] Räisänen, U. , and K. Hunt . 2014. “The Role of Gendered Constructions of Eating Disorders in Delayed Help‐Seeking in Men: A Qualitative Interview Study.” BMJ Open 4: e004342. 10.1136/bmjopen-2013-004342. PMC3987710 24713213

[eat24526-bib-0033] Ralph, A. F. , L. Brennan , S. Byrne , et al. 2022. “Management of Eating Disorders for People with Higher Weight: Clinical Practice Guideline.” Journal of Eating Disorders 10, no. 1: 121. 10.1186/s40337-022-00622-w. 35978344 PMC9386978

[eat24526-bib-0027] Rizvi, S. L. , C. B. Peterson , S. J. Crow , and W. S. Agras . 2000. “Test‐Retest Reliability of the Eating Disorder Examination.” International Journal of Eating Disorders 28, no. 3: 311–316. 10.1002/1098-108X(200011)28:3<311::AID-EAT8>3.0.CO;2-K. 10942917

[eat24526-bib-0028] Schmidt, R. , T. Kirsten , A. Hiemisch , W. Kiess , and A. Hilbert . 2019. “Interview‐Based Assessment of Avoidant/Restrictive Food Intake Disorder (ARFID): A Pilot Study Evaluating an ARFID Module for the Eating Disorder Examination.” International Journal of Eating Disorders 52, no. 4: 388–397. 10.1002/eat.23063. 30843618

[eat24526-bib-0029] Strother, E. , R. Lemberg , S. C. Stanford , and D. Turberville . 2012. “Eating Disorders in Men: Underdiagnosed, Undertreated, and Misunderstood.” Eating Disorders 20, no. 5: 346–355. 10.1080/10640266.2012.715512. 22985232 PMC3479631

[eat24526-bib-0030] Tomiyama, A. J. , D. Carr , E. M. Granberg , et al. 2018. “How and Why Weight Stigma Drives the Obesity ‘Epidemic’ and Harms Health.” BMC Medicine 16, no. 1: 123. 10.1186/s12916-018-1116-5. 30107800 PMC6092785

[eat24526-bib-0031] Weissman, R. S. , A. E. Becker , C. M. Bulik , et al. 2016. “Speaking of That: Terms to Avoid or Reconsider in the Eating Disorders Field.” International Journal of Eating Disorders 49, no. 4: 349–353. 10.1002/eat.22528. 27084795

