# Supplementary material for: Reflections on Using the Eating Disorders Examination to Assess Eating Disorder Pathology in Queer Men
Source: Int J Eat Disord. 2025 Aug 13;58(11):2157–66. doi: 10.1002/eat.24526 (PMC12605624; doi:10.1002/eat.24526)
Supplement: Supplementary file 1 — Data S1: Supporting Information. [file EAT-58-2157-s001.docx]

**Supplementary Materials: Interviewer reflection prompts**

**Note:** interviewers were provided the below examples questions for reflection, but were encouraged to use these loosely and reflect outside of these constraints.

**Scope of paper**: Insights from clinical eating disorder (EDE) interviews with queer cis men

**Example questions for reflection:**

1. What do you want other clinicians to know about using the EDE to assess ED pathology in queer cis men?
2. What are the benefits/positives of using the EDE to assess ED pathology in queer cis men?
   1. E.g., was it particularly useful for delving into a particular type of symptomatology?
   2. Anything else particularly useful about the protocol? Specific examples?
3. What challenges did you encounter with the EDE? For example:
   1. Were there instances where you felt like you wanted to delve deeper into a particular experience/symptom but were unable to?
   2. Any other aspects of the interview protocol you found particularly challenging?
   3. Are there any particular EDE questions that are clunky / sensitive / badly worded that you think could be altered/removed?
   4. Think of an interview you may have found particularly challenging. Can you give specific examples (without revealing any identifying information) of what you found challenging?
4. How comprehensive do you feel the EDE is for assessing ED symptoms for this particular group?
5. [For interviewers with experience using EDE in other populations – e.g., women and/or heterosexual men] Were there any unique considerations / challenges when using EDE for queer men versus others (e.g., women?)
   1. How has your experience interviewing for this study differed (if at all) to your experience using the EDE for other purposes/in other groups
6. How has your experience changed over the course of doing more interviews?
   1. E.g., any changes in your experience with parts of the protocol you previously found challenging?
